# Antibiotic Prescribing Patterns in Hospitalized Pediatric Patients With Clinically Suspected Enteric Fever: A Descriptive Study

**DOI:** 10.7759/cureus.103186

**Published:** 2026-02-08

**Authors:** Gulam Abdul Kadir Khan, Muhammad Arslan Siddiqui, Dheeraj Bansal, Jagan Nadipelly, Hardat Persaud

**Affiliations:** 1 Medicine, Texila American University, Georgetown, GUY; 2 Public Health Sciences, Texila American University, Georgetown, GUY; 3 Pharmacology, Texila American University, Georgetown, GUY; 4 Pediatrics, Woodlands Hospital, Georgetown, GUY

**Keywords:** antibiotic prescribing patterns, ceftriaxone, empirical antibiotic therapy, hospitalized children, pediatric enteric fever

## Abstract

Background: Enteric fever remains an important cause of morbidity among children in developing regions and is frequently managed with empirical antimicrobial therapy. In the setting of evolving antimicrobial resistance and diagnostic limitations, describing real-world antibiotic prescribing practices in pediatric patients is essential. This study aimed to describe antibiotic prescribing patterns among hospitalized pediatric patients with clinically suspected enteric fever and to explore their distribution across clinical and laboratory parameters.

Methods: A hospital-based retrospective observational study was conducted among 100 hospitalized pediatric patients (>2 years of age) clinically diagnosed with enteric fever. Demographic details, clinical features, vaccination status, laboratory parameters (hemoglobin, total leukocyte count, erythrocyte sedimentation rate, platelet count, C-reactive protein, and Widal test results), and treatment details were extracted from medical records. Antibiotic utilization patterns involving injectable ceftriaxone, injectable ofloxacin, injectable amikacin, and oral doxycycline were analyzed using descriptive statistics. Associations between antibiotic use and selected clinical and laboratory variables were explored using the chi-square test, with a p-value ≤0.05 considered statistically significant.

Results: The mean age of patients was 6.03±3.08 years, with a near-equal gender distribution. Fever was the most common presenting symptom (90%), followed by vomiting (45%) and abdominal pain (23%). Laboratory evaluation revealed mild anemia and elevated inflammatory markers in a proportion of patients. Injectable ceftriaxone was the most frequently prescribed antibiotic (83%), followed by oral doxycycline (27%), injectable ofloxacin (26%), and injectable amikacin (11%). Variations in antibiotic use were observed across different clinical features, laboratory parameters, and treatment intensity. Age did not show a statistically significant association with antibiotic selection.

Conclusion: This study describes prevailing antibiotic prescribing patterns among hospitalized pediatric patients with clinically suspected enteric fever. Injectable ceftriaxone was the most commonly used empirical antibiotic, while other agents were prescribed selectively. The findings highlight existing prescribing trends in inpatient pediatric care and underscore the need for improved microbiological confirmation to better inform antibiotic selection in enteric fever.

## Introduction

Enteric fever, caused by *Salmonella enterica* serovars Typhi and Paratyphi, continues to pose a significant public health challenge, particularly among pediatric populations in endemic regions characterized by inadequate sanitation and limited access to safe drinking water [1,2]. Despite ongoing efforts to improve hygiene and water safety, children remain disproportionately affected and frequently present with prolonged fever, gastrointestinal symptoms, and systemic inflammatory responses that complicate early clinical diagnosis and management [3,4]. The burden of pediatric enteric fever is further compounded by school absenteeism, repeated hospital visits, and an increased risk of complications when diagnosis or treatment is delayed [5]. Studies from sub-Saharan Africa have also demonstrated a substantial burden of pediatric typhoid fever, highlighting its public health relevance beyond South Asia.

The therapeutic management of enteric fever has evolved considerably over recent decades due to the emergence and spread of antimicrobial resistance. Conventional first-line agents such as chloramphenicol, ampicillin, and cotrimoxazole have been largely replaced in many settings because of widespread resistance, leading to increased reliance on fluoroquinolones, third-generation cephalosporins, and macrolides [6-8]. Surveillance studies have documented rising resistance to fluoroquinolones and reduced susceptibility to cephalosporins, along with the emergence of multidrug-resistant and extensively drug-resistant *Salmonella* strains, including among pediatric populations [9-12]. These trends highlight challenges in empirical treatment selection, particularly in settings where microbiological confirmation is limited and clinical diagnosis may overestimate true disease burden [13-15].

Considerable variability in antibiotic prescribing practices has been reported across healthcare institutions and geographic regions, especially with respect to the choice of parenteral versus oral therapy and the use of combination regimens in children with varying disease severity [16,17]. In routine clinical practice, laboratory parameters such as hemoglobin levels, inflammatory markers, platelet counts, and serological test results are frequently assessed; however, their role in influencing antibiotic selection in pediatric enteric fever has not been well described [18-20]. Previous studies have shown that several clinical and laboratory factors may influence blood culture positivity in children, further complicating diagnostic interpretation and treatment decisions [21].

Global disease burden estimates continue to identify typhoid and paratyphoid fevers as important contributors to pediatric morbidity in endemic regions [21,22]. Vaccination has emerged as a key preventive strategy, with typhoid conjugate vaccines demonstrating favorable efficacy and immunogenicity profiles in both clinical trials and population-based studies, leading to updated international recommendations for their use in endemic settings [23-26]. Nevertheless, epidemiological studies and meta-analyses indicate that pediatric enteric fever remains prevalent in many low- and middle-income countries [27-30].

Recent genomic studies have further highlighted the emergence and international spread of extensively drug-resistant *S. enterica* serovar Typhi strains with resistance to fluoroquinolones and third-generation cephalosporins [31-34]. Ongoing surveillance data confirm the continued incidence of pediatric enteric fever in South Asia and other comparable endemic regions [35]. In addition, healthcare utilization patterns and surveillance methodologies have been shown to influence observed disease incidence and treatment practices in endemic settings [36]. In this context, the present study aimed to describe antibiotic prescribing patterns among hospitalized pediatric patients with clinically suspected enteric fever and to examine their distribution across clinical features and laboratory parameters in a tertiary care hospital setting [36-38]. By characterizing real-world prescribing practices, this study seeks to provide insight into current inpatient management trends in resource-limited, endemic settings [39-48].

## Materials and methods

Study design and setting

This descriptive observational study was conducted in the Department of Pediatrics at a tertiary care hospital (Woodlands Hospital, Georgetown, Guyana), South America. Routinely collected clinical records were reviewed over an eight-month period from March 2025 to October 2025. The study included hospitalized pediatric patients who were clinically diagnosed with enteric fever and managed according to standard institutional clinical practice. The primary objective was to describe antibiotic prescribing patterns in pediatric enteric fever and to examine their distribution across selected clinical and laboratory parameters.

Study population

The study population comprised pediatric inpatients aged two to 16 years with a clinical diagnosis of enteric fever during the study period. A total of 100 consecutive eligible patients (n=100) meeting the predefined inclusion criteria were included in the analysis. Children below two years of age were excluded because they are managed under separate diagnostic and therapeutic protocols due to differences in clinical presentation and antimicrobial dosing considerations.

Inclusion and exclusion criteria

Children aged two to 16 years who were clinically diagnosed with enteric fever, required hospitalization, received antimicrobial therapy for suspected enteric fever, and had complete clinical and laboratory records available were included.

Patients were excluded if they were adults (>16 years), did not have a diagnosis of enteric fever, had incomplete or missing clinical or laboratory data, or were transferred from other healthcare facilities after initiation of antibiotic therapy. Outpatient cases were not included due to variability in documentation and lack of consistent follow-up.

Diagnostic criteria

The diagnosis of enteric fever was based on clinical suspicion supported by routine laboratory investigations, consistent with standard practice in endemic settings. Microbiological confirmation by blood culture or molecular testing was not uniformly available due to prior antibiotic exposure and resource limitations. As a result, antibiotic therapy was initiated empirically based on clinical judgment and available laboratory findings, reflecting real-world inpatient management practices in resource-limited settings. Serological testing using the Widal test was performed as part of routine evaluation in a proportion of patients.

Data collection

Data were extracted retrospectively from hospital medical records using a structured data collection format. Variables recorded included demographic characteristics, clinical presentation, vaccination status, and laboratory parameters such as hemoglobin, total leukocyte count, erythrocyte sedimentation rate (ESR), platelet count, C-reactive protein, and Widal test results. Details of antibiotic therapy, including the use of injectable ceftriaxone, injectable ofloxacin, injectable amikacin, and oral doxycycline, were documented. Duration of antibiotic treatment and length of hospital stay were also recorded.

Statistical analysis

Collected data were reviewed and analyzed using IBM SPSS Statistics (Armonk, NY). Categorical variables were expressed as frequencies and percentages (n(%)). Continuous laboratory parameters were categorized into clinically relevant groups to facilitate categorical analysis. Associations between antibiotic prescribing patterns and selected clinical or laboratory parameters were explored using the chi-square test, with a p-value of ≤0.05 considered statistically significant.

Ethical considerations

The study involved retrospective analysis of existing, anonymized hospital medical records and did not include any experimental intervention, prospective enrollment, or modification of standard patient management. In accordance with institutional policy, Institutional Review Board (IRB)/Ethics Committee approval was waived by the Institutional Review Board/Ethics Committee of Woodlands Hospital Ltd., Georgetown, Guyana, as documented in the institutional ethics waiver letter. The requirement for informed consent was waived due to the retrospective nature of the study and the use of anonymized data. Patient confidentiality was strictly maintained, and the study was conducted in accordance with the ethical principles of the Declaration of Helsinki.

## Results

Study population and demographic characteristics

**Figure 1:**
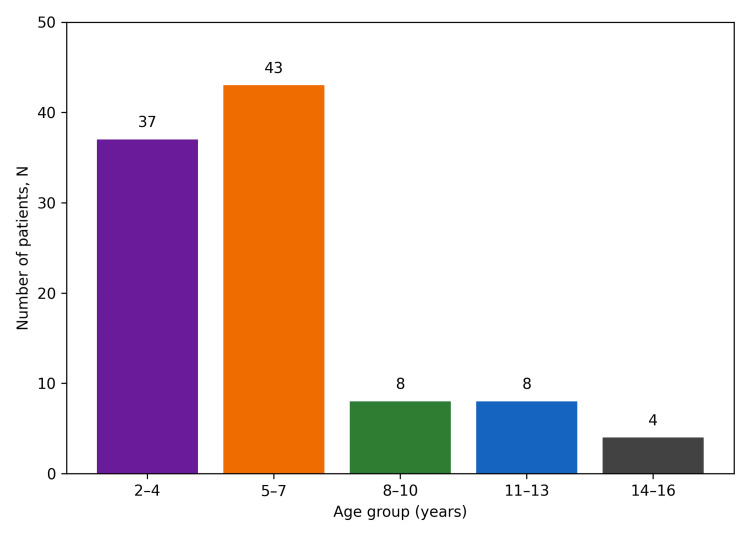
Age distribution of pediatric patients with enteric fever (n=100)

A total of 100 hospitalized pediatric patients with clinically suspected enteric fever were included in the study. The mean age of the study population was 6.03±3.08 years. The largest proportion of patients belonged to the five- to seven-year age group (43%), followed by children aged two to four years (37%). Patients aged eight to 10 years and 11-13 years each accounted for 8% of cases, while those aged 14-16 years constituted 4% of the study population. The gender distribution was nearly equal, with 51% males and 49% females (Figure 1).

Clinical presentation and vaccination status

**Figure 2:**
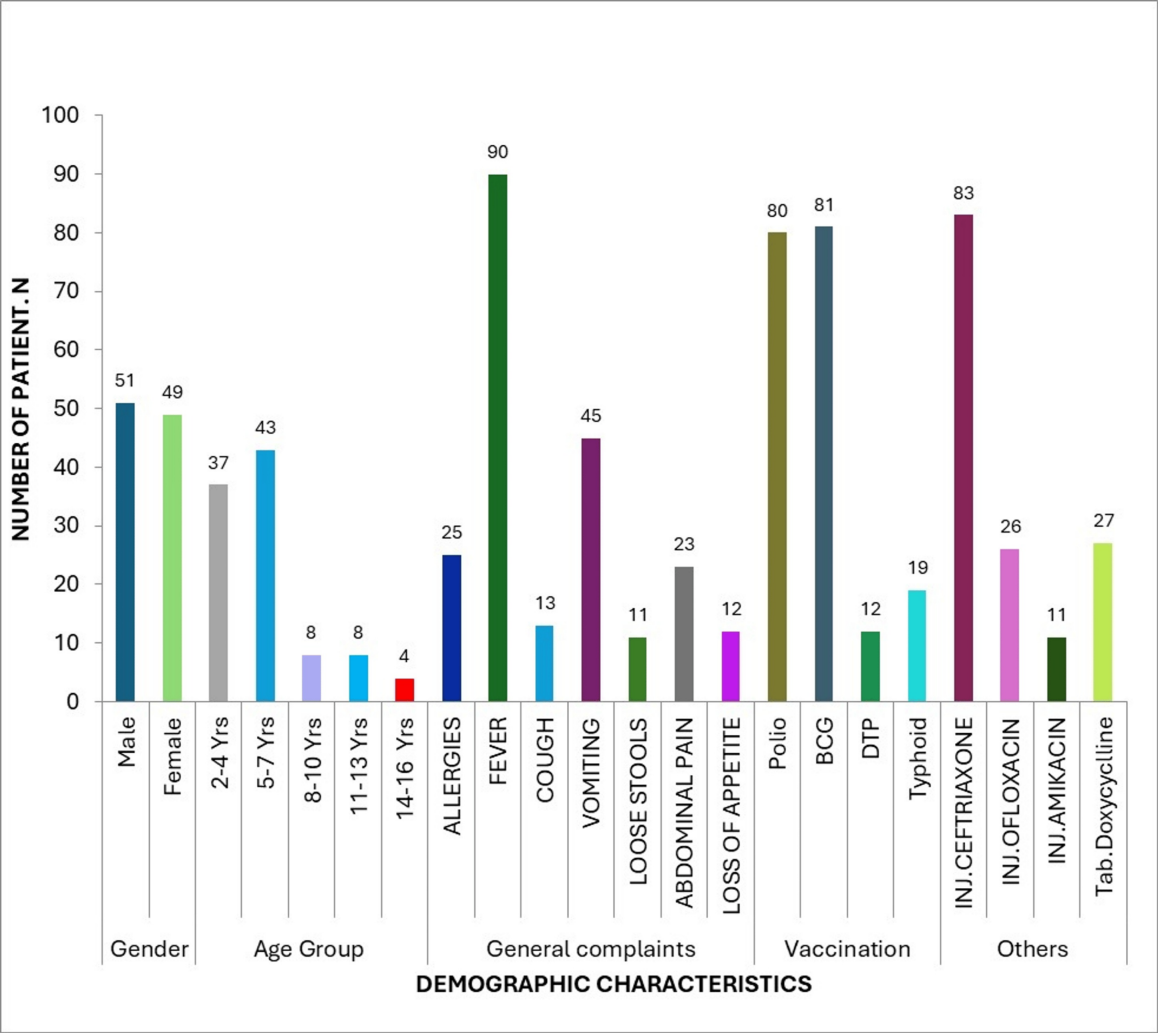
Demographic characteristics, clinical presentation, vaccination status, and antibiotic use among pediatric enteric fever patients (n=100)

Fever was the most frequently reported presenting symptom, observed in 90% of patients. Other clinical features included cough (13%), abdominal pain (23%), vomiting (45%), loss of appetite (12%), and loose stools (11%). Regarding immunization history, bacillus Calmette-Guérin (BCG) vaccination was documented in 81% of patients, polio vaccination in 80%, diphtheria-tetanus-pertussis (DTP) vaccination in 12%, and typhoid vaccination in 19% (Figure 2).

Antibiotic prescribing patterns

**Figure 3:**
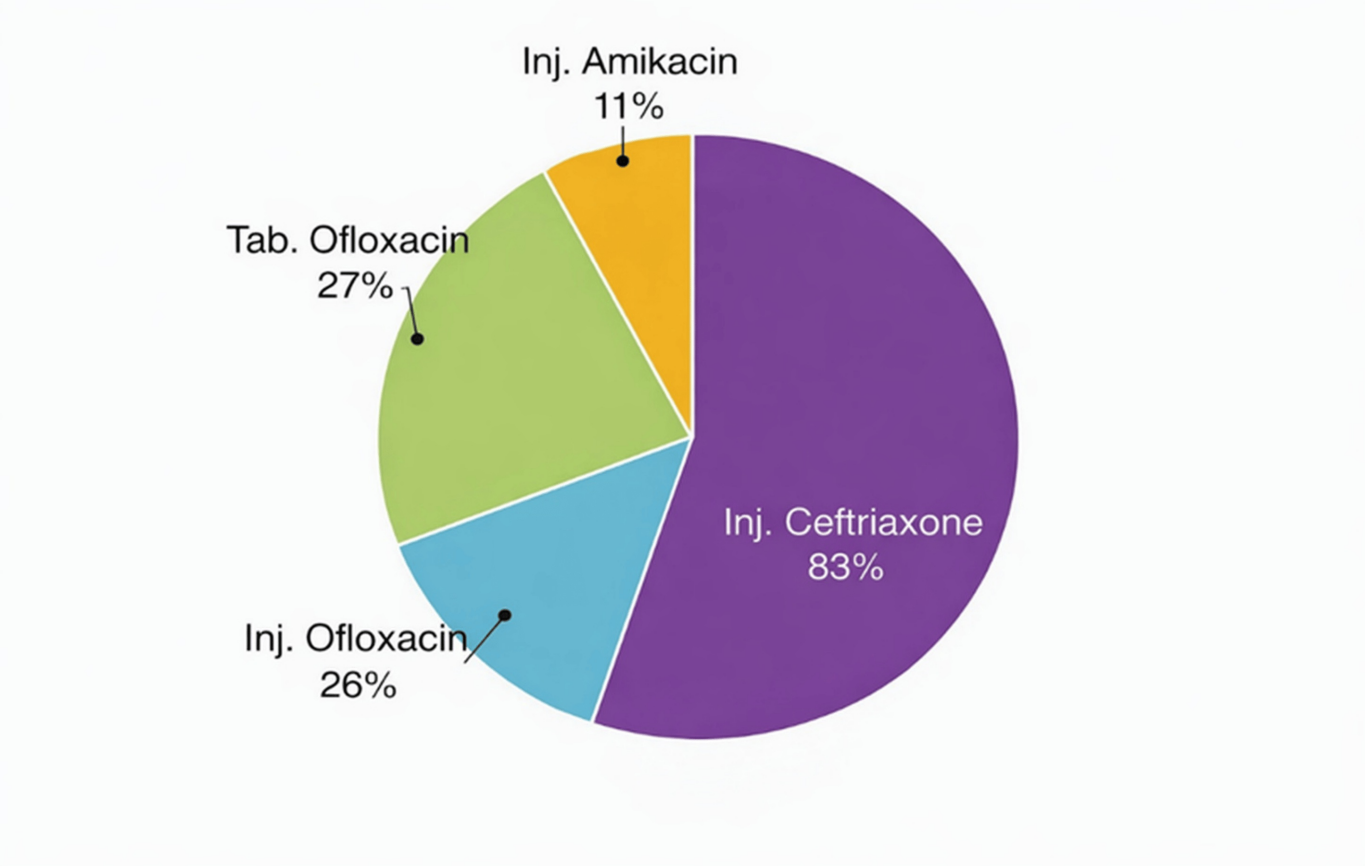
Antibiotic utilization pattern among pediatric enteric fever patients (n=100)

Injectable ceftriaxone was the most frequently prescribed antibiotic, administered to 83% of patients. Oral doxycycline was prescribed in 27%, injectable ofloxacin in 26%, and injectable amikacin in 11% of patients. More than one antibiotic was prescribed in several patients, reflecting escalation of therapy based on clinical severity and laboratory findings. Percentages may therefore exceed 100% due to overlapping antibiotic use (Figure 3).

Associations between antibiotic use and clinical or laboratory parameters

Significant associations were observed between antibiotic use and several clinical and laboratory parameters. Injectable amikacin use was significantly associated with elevated erythrocyte sedimentation rate (ESR) (p≤0.05), platelet abnormalities, and positive Widal test results (p≤0.05). Oral doxycycline use demonstrated significant associations with gastrointestinal symptoms, prolonged hospital stay, and higher treatment intensity (p≤0.05). Patient age was not significantly associated with antibiotic selection (p>0.05).

Associations between antibiotic use and clinical, laboratory, and treatment variables

Associations between antibiotic prescribing patterns and selected demographic, clinical, laboratory, and treatment-related variables were explored using the chi-square test. The results of these analyses are summarized in Table 1 and Table 2.

**Table 1:** Association of antibiotic use with demographic and clinical variables in hospitalized pediatric patients with clinically suspected enteric fever (n=100) Values represent χ² (p-value). A p-value ≤0.05 was considered statistically significant.

Variable	Sub-variable	No. of patients (N=100)	Ceftriaxone, χ²	Ceftriaxone, p	Ofloxacin, χ²	Ofloxacin, p	Amikacin, χ²	Amikacin, p	Doxycycline, χ²	Doxycycline, p
Age (years)	2-4	37	0.506	0.973	3.343	0.502	3.163	0.531	3.378	0.497
Age (years)	5-7	43	0.506	0.973	3.343	0.502	3.163	0.531	3.378	0.497
Age (years)	8-10	8	0.506	0.973	3.343	0.502	3.163	0.531	3.378	0.497
Age (years)	11-13	8	0.506	0.973	3.343	0.502	3.163	0.531	3.378	0.497
Age (years)	14-16	4	0.506	0.973	3.343	0.502	3.163	0.531	3.378	0.497
Gender	Male	51	3.145	0.056	0.114	0.736	4.697	0.03	3.633	0.055
Gender	Female	49	3.145	0.056	0.114	0.736	4.697	0.03	3.633	0.055
Vomiting	Present	45	9.141	0.002	4.639	0.031	6.439	0.011	10.48	0.001
Loose stools	Present	11	0.548	0.459	0.01	0.919	0.046	0.83	8.417	0.004
Abdominal pain	Present	23	0.003	0.955	7.278	0.007	1.35	0.245	2.952	0.056
Loss of appetite	Present	12	23.84	0.0001	7.41	0.006	13.099	0.0001	3.66	0.054

**Table 2:** Association of antibiotic use with laboratory and treatment-related variables in hospitalized pediatric patients with clinically suspected enteric fever (n=100) Values represent χ² (p-value). A p-value ≤0.05 was considered statistically significant.

Variable	Sub-variable	No. of patients (N=100)	Ceftriaxone, χ²	Ceftriaxone, p	Ofloxacin, χ²	Ofloxacin, p	Amikacin, χ²	Amikacin, p	Doxycycline, χ²	Doxycycline, p
Temperature (°C)	37-38	37	0.916	0.633	11.921	0.003	5.28	0.051	6	0.05
Temperature (°C)	39-40	59	0.916	0.633	11.921	0.003	5.28	0.051	6	0.05
Temperature (°C)	41-42	4	0.916	0.633	11.921	0.003	5.28	0.051	6	0.05
Hemoglobin (g/dL)	6-8	26	8.913	0.012	41.138	0.0001	5.902	0.052	29.119	0.0001
Hemoglobin (g/dL)	9-11	62	8.913	0.012	41.138	0.0001	5.902	0.052	29.119	0.0001
Hemoglobin (g/dL)	12-14	12	8.913	0.012	41.138	0.0001	5.902	0.052	29.119	0.0001
Total leukocyte count	3000-6000	66	5.355	0.051	18.1	0.0001	2.33	0.312	7.209	0.027
Total leukocyte count	7000-10000	23	5.355	0.051	18.1	0.0001	2.33	0.312	7.209	0.027
Total leukocyte count	11000-13000	11	5.355	0.051	18.1	0.0001	2.33	0.312	7.209	0.027
Platelet count	Abnormal	30	1.218	0.27	0.01	0.921	10.745	0.001	2.032	0.154
Platelet count	Normal	70	1.218	0.27	0.01	0.921	10.745	0.001	2.032	0.154
Widal test	Positive	100	11.53	0.003	1.928	0.381	9.324	0.009	4.992	0.052
Hospital stay (days)	2-3	46	0.192	0.661	1.833	0.176	0.462	0.497	8.418	0.004
Hospital stay (days)	4-6	54	0.192	0.661	1.833	0.176	0.462	0.497	8.418	0.004
Number of drugs prescribed	3-5	63	11.279	0.004	22.395	0.0001	5.313	0.07	63.038	0.0001
Number of drugs prescribed	6-7	27	11.279	0.004	22.395	0.0001	5.313	0.07	63.038	0.0001
Number of drugs prescribed	8-9	10	11.279	0.004	22.395	0.0001	5.313	0.07	63.038	0.0001
Number of antibiotics used	1	60	15.781	0.001	55.886	0.0001	35.819	0.0001	63.28	0.0001
Number of antibiotics used	2	16	15.781	0.001	55.886	0.0001	35.819	0.0001	63.28	0.0001
Number of antibiotics used	3	20	15.781	0.001	55.886	0.0001	35.819	0.0001	63.28	0.0001
Number of antibiotics used	4	4	15.781	0.001	55.886	0.0001	35.819	0.0001	63.28	0.0001
Injections per day	1	8	2.107	0.717	9.368	0.053	6.194	0.185	23.42	0.0001
Injections per day	2	18	2.107	0.717	9.368	0.053	6.194	0.185	23.42	0.0001
Injections per day	3	33	2.107	0.717	9.368	0.053	6.194	0.185	23.42	0.0001
Injections per day	4	34	2.107	0.717	9.368	0.053	6.194	0.185	23.42	0.0001
Injections per day	5	7	2.107	0.717	9.368	0.053	6.194	0.185	23.42	0.0001

## Discussion

The present study described antibiotic prescribing patterns among hospitalized pediatric patients with clinically suspected enteric fever and explored their distribution across selected clinical and laboratory parameters in a tertiary care setting. The findings demonstrate a predominantly empirical prescribing approach, with injectable ceftriaxone emerging as the most frequently used antibiotic, followed by oral doxycycline, injectable ofloxacin, and injectable amikacin. This pattern is consistent with current real-world practice in endemic regions, where third-generation cephalosporins are commonly preferred because of their favorable safety profile, broad antimicrobial coverage, and increasing resistance to traditional first-line agents.

A key observation of this study was the presence of statistically significant associations between antibiotic selection and routinely assessed clinical and laboratory parameters, including body temperature, hemoglobin level, total leukocyte count, ESR, platelet count, and Widal test results. These findings suggest that clinicians rely heavily on indicators of clinical severity and basic hematological investigations when selecting antimicrobial therapy, particularly in the absence of consistent microbiological confirmation. Such an approach reflects pragmatic decision-making in resource-limited settings, where culture or molecular diagnostics may be unavailable or compromised by prior antibiotic exposure.

Hemoglobin level demonstrated multiple significant associations with antibiotic use, indicating that anemia may be perceived as a marker of disease severity or prolonged illness, prompting escalation or intensification of therapy. Injectable ofloxacin showed significant associations with elevated temperature, hemoglobin level, total leukocyte count, and ESR, suggesting its selective use in patients with persistent fever or higher inflammatory burden. This observation is consistent with published literature in which fluoroquinolones are often reserved for cases with inadequate response to initial therapy, despite growing concerns regarding antimicrobial resistance.

Injectable amikacin was prescribed in a smaller proportion of patients and demonstrated significant associations primarily with platelet abnormalities and Widal positivity. This pattern supports its role as a reserve or adjunct agent, likely employed in more severe or complicated clinical scenarios rather than for routine empirical use. Oral doxycycline showed associations with hematological parameters and treatment-related factors, including longer hospital stay, which may reflect its use in selected cases such as prolonged illness or as part of step-down oral therapy following initial parenteral treatment.

Although Widal test results demonstrated statistical associations with antibiotic selection, these findings must be interpreted cautiously. The Widal test has well-recognized limitations in endemic settings, including low specificity and cross-reactivity, which may lead to false-positive results. Accordingly, the observed associations likely reflect clinicians’ reliance on available diagnostic tools rather than true etiological confirmation. This underscores the importance of cautious interpretation of serological results and highlights the ongoing need for improved access to reliable microbiological diagnostics in endemic regions.

Notably, C-reactive protein did not show a significant association with antibiotic prescribing decisions, suggesting that clinicians may prioritize readily available hematological parameters and overall clinical assessment over inflammatory biomarkers when making treatment decisions. Overall, the associations observed in this study reflect prescribing behavior and clinical decision-making patterns rather than causal relationships between laboratory abnormalities and antibiotic efficacy.

Confounding factors

Several potential confounding factors may have influenced antibiotic prescribing decisions in this study. Disease severity at presentation, including duration and intensity of fever, systemic involvement, and overall clinical stability, likely played an important role in determining the choice and intensity of antimicrobial therapy. However, standardized severity scoring systems were not uniformly documented and could not be accounted for in the analysis.

Prior antibiotic exposure before hospital admission may have influenced both clinical presentation and subsequent prescribing patterns, as pretreatment can alter laboratory parameters and perceived treatment response. In addition, comorbid conditions and nutritional status, which may affect clinician decision-making and antibiotic escalation, were not consistently available in the medical records. Vaccination status, particularly receipt of typhoid conjugate vaccine, may also have modified disease severity and laboratory findings, indirectly influencing antibiotic selection. Furthermore, duration of hospitalization and individual clinician preference may have contributed to step-up or step-down therapy decisions independent of baseline clinical or laboratory variables.

Because of the study design and the absence of multivariable regression analysis, these potential confounders could not be adjusted for and may have influenced the observed associations between antibiotic choice and clinical or laboratory parameters. Consequently, the findings should be interpreted as descriptive of real-world prescribing behavior rather than indicative of causal relationships.

Limitations

This study has several limitations. The retrospective observational design limits the ability to infer causal relationships between clinical or laboratory parameters and antibiotic selection. Microbiological confirmation of enteric fever was not uniformly available, and diagnoses were primarily based on clinical assessment supported by routine laboratory findings, reflecting real-world practice in endemic settings. The Widal test, while commonly used, has known limitations related to specificity and cross-reactivity; therefore, associations involving Widal results should be interpreted with caution.

Continuous laboratory variables were categorized to permit chi-square analysis, which may have resulted in some loss of information. In addition, multivariable regression analysis was not performed, limiting adjustment for potential confounders such as disease severity, prior antibiotic exposure, comorbidities, vaccination status, and duration of hospitalization. Finally, as this was a single-center study, the generalizability of the findings may be limited.

## Conclusions

This study describes antibiotic prescribing patterns among hospitalized pediatric patients with clinically suspected enteric fever and their distribution across clinical presentation and routinely assessed laboratory parameters. Injectable ceftriaxone was the most commonly prescribed empirical antibiotic, while ofloxacin, amikacin, and doxycycline were used selectively in patients with indicators of greater clinical severity or laboratory abnormalities. Observed associations with body temperature, hemoglobin level, total leukocyte count, ESR, platelet count, and Widal test results reflect real-world clinical decision-making in settings where microbiological confirmation is limited. These findings provide insight into current inpatient prescribing practices and underscore the importance of strengthening diagnostic capacity to better inform antibiotic selection in pediatric enteric fever.
